# Unconventional specular optical rotation in the charge ordered state of Kagome metal CsV_3_Sb_5_

**DOI:** 10.1038/s41467-023-41080-5

**Published:** 2023-09-01

**Authors:** Camron Farhang, Jingyuan Wang, Brenden R. Ortiz, Stephen D. Wilson, Jing Xia

**Affiliations:** 1 Department of Physics and Astronomy, University of California, Irvine, CA 92697 USA; 2 Materials Department, University of California, Santa Barbara, Santa Barbara, CA 93106 USA

**Keywords:** Magnetic properties and materials, Characterization and analytical techniques

## Abstract

Kagome metals AV_3_Sb_5_ (A = K, Cs, Rb) provide a rich platform for intertwined orders, where evidence for time-reversal symmetry breaking, likely due to the long-sought loop currents, has emerged in STM and muon spin relaxation experiments. An isotropic component in the spontaneous optical rotation has also been reported and was interpreted as the magneto-optic Kerr effect. Intriguingly, the observed rotations differ by five orders of magnitude between different wavelengths and samples, suggesting more intricate physics. Here we report optical rotation and polar Kerr measurements in CsV_3_Sb_5_ crystals at the same wavelength. We observe large isotropic components of 1 milliradian in the optical rotation that do not respond to applied magnetic fields, while the spontaneous Kerr signal is less than 20 nanoradians. Our results prove unambiguously that the reported isotropic rotation is not from time-reversal symmetry breaking but represents the long-sought specular optical rotation and indicates a new intertwined order.

## Abstract (web summary)

Kagome materials, such as CsV3Sb5, a rich array of correlated phase, including a time-reversal symmetry breaking phase, which could possibly be the result of loop currents. Attempts to verify this with magneto-optical measurements have yielded mixed results. Here, Farhang et al show that the magneto-optical signals are due to specular optical rotation. ‘

## Introduction

The Kagome lattice is a rich platform for novel phases of matter due to the interplay between strong correlation and topological orders, such as in the case of Chern topological magnet TbMn_6_Sn_6_^1^. In particular the recently discovered quasi-two-dimensional Kagome compounds AV_3_Sb_5_ (A = K, Rb and Cs)^2,3^ have elaborate phase diagrams due to the ideal Kagome network governed by layers of vanadium and antimony intercalated by alkali metal ions^2,3^. The fascinating electronic band structure containing Dirac cones, flat bands, and Van Hove singularities^3,4^ leads to intertwined^5^ electronic instabilities and exhibits charge density wave (CDW)^6–10^, pressure-tunable superconductivity^3,11–14^, and time reversal symmetry breaking (TRSB) that has been revealed by STM^15^ and muon-spin relaxation (µSR)^16–18^ experiments. The TRSB state may be related to the long-sought loop currents^19,20^, and could produce a spontaneous magneto-optic Kerr effect (MOKE)^21,22^ rotation *θ*_*K*_, which arises from the optical phase difference ∆*φ* = 2θ*K* between counter-propagating circularly polarized light beams that are time-reversal images of each other.

Indeed, two optical rotation experiments performed at 800 nm wavelength have revealed^23,24^ a spontaneous total rotation *θ*_*T*_ in the form of *θ*_*T*_ = *θ*_*C*_ + *θ*_*P*_ sin(2*α* − *A*), where *α* is the incident polarization angle, and *A* is the principle axis direction. *θ*_*P*_ represents the anisotropic component due to the nematicity^23,24^ of the CDW state. Since translational symmetry is also broken, it will be called “anisotropy” in this work to avoid confusion. *θ*_*C*_ represents the isotropic (polarization independent) component and was found to be 0.5 mrad^24^ and 50 μrad^23^ in CsV_3_Sb_5_. In transmission, such an isotropic *θ*_*C*_ component could arise from optical activities in a chiral material, which causes an optical phase difference between circularly polarized lights propagating in the same direction. However, this effect is usually rejected^25^ in normal incidence reflection used in the above experiments. Therefore, the observed isotropic rotation *θ*_*C*_ has been naturally attributed to a spontaneous MOKE signal *θ*_*K*_ due to TRSB at *T*_*CDW*_^23,24^. Intriguingly, it is noted^24^ that such a large *θ*_*C*_ can be compared with that of some magnetic materials, while both µSR^16–18^ experiments and theoretical calculations^26^ indicate extremely small sub-gauss level magnetic flux density that would usually lead to nano-radians to sub-microradian levels of spontaneous MOKE signals^27,28^. Dedicated MOKE experiments^29–31^ have been carried out in CsV_3_Sb_5_ at 1550 nm wavelength utilizing zero-loop Sagnac interferometers^32^ in an attempt to resolve this puzzle. Such an interferometer is sensitive only to TRSB effects by detecting the optical phase difference ∆*φ* between time-reversed counter-propagating circularly polarized light beams, which is twice the MOKE angle: ∆*φ* = 2*θ*_*K*_. Surprisingly the 1550 nm MOKE experiments report either a much smaller spontaneous Kerr signal *θ*_*K*_ ~ 2 μrad^30^ or near-zero values *θ*_*K*_ < 0.03 μrad^29,31^. To explain this giant discrepancy, one is tempted to assume a resonance enhancement at 800 nm wavelength over 1550 nm. On the contrary the near-infrared spectra of CsV_3_Sb_5_ are rather flat^33,34^ with a Lorentz resonance at 6000 cm^−1^ (equivalent to 1667 nm)^34^ suggesting instead a larger expected signal at the 1550 nm wavelength.

To solve this outstanding mystery, we perform optical rotation and polar MOKE measurements at 1550 nm wavelength on the same CsV_3_Sb_5_ crystals. Rather surprisingly, we observe giant *θ*_*C*_ ~ ±1200 μrad below *T*_*CDW*_ but negligible *θ*_*K*_ <0.02 *μrad* at zero magnetic fields. The *θ*_*C*_ component does not respond to applied magnetic fields, which is opposite to MOKE and to the magnetic field responses found in STM^15^ and µSR^16–18^ experiments. Therefore, we conclude that the observed *θ*_*C*_ represents an unconventional optical rotation that is not due to either TRSB, anisotropy or chiral order, but originates from a new intertwined order that onsets at *T*_*CDW*_.

## Results

### CDW transition

**Fig. 1:**
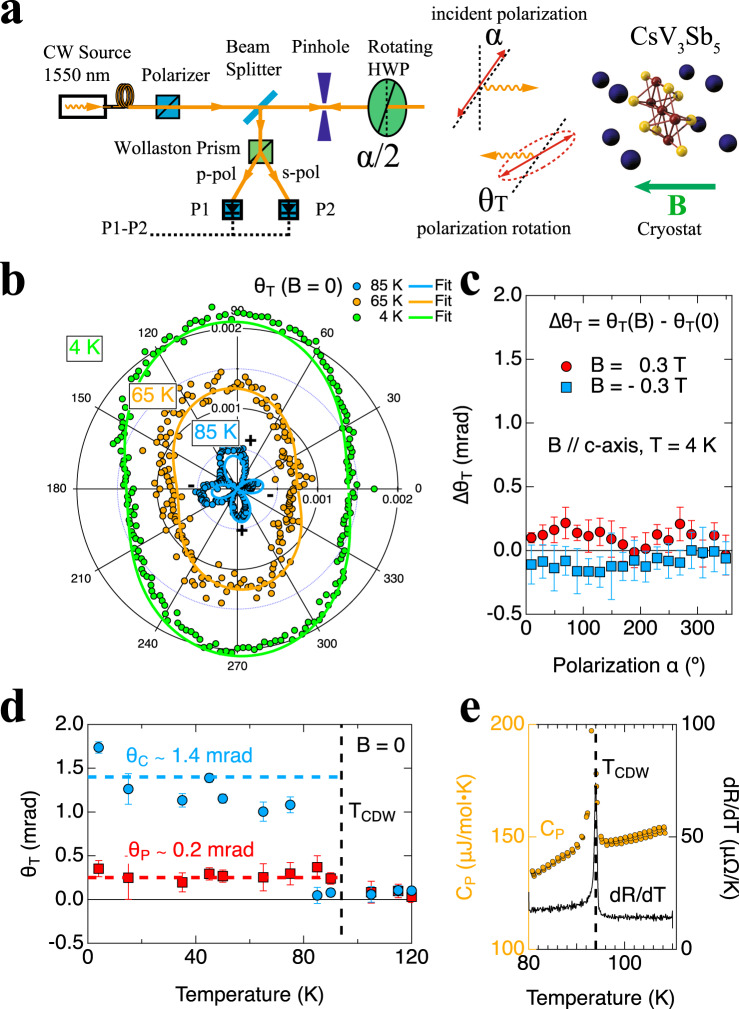
Optical rotation *θ*_*T*_ of CsV3Sb5 sample 1. **a** Polarization rotation *θ*_*T*_ at normal incidence is measured as a function of incident polarization angle *α*. See SI for details. **b** Polar plots of spontaneous *θ*_*T*_ at representative temperatures, fitted with *θ*_*T*_ = *θ*_*C*_ + *θ*_*P*_ sin(2*α* − *A*). See Fig. S3a–l for full data. **c** Changes of rotation ∆*θ*_*T*_ by ±0.3 *T* magnetic fields at 4 *K*, showing opposite magnetic fields fail to flip the sign of *θ*_*C*_, where error bars represent standard deviation. **d** Fitted *θ*_*C*_ (*θ*_*P*_) up to 1.4 *mrad* (0.2 mrad) with onsets below *T*_*CDW*_, where error bars represent uncertainty in fitting. **e** *T*_*CDW*_ ~94 *K* is marked by sharp peaks in the specific heat *C*_*P*_ and resistance derivative *dR*/*dT*.

High quality CsV_3_Sb_5_ single crystals, dubbed sample 1 and 2, were grown by the self-flux method at UCSB^3^. The first order CDW transition at *T*_*CDW*_ ~ 94 *K* is clearly characterized by sharp peaks in the heat capacity *C*_*P*_ and resistance derivative *dR*/*dT* as shown in Fig. 1e. The crystals were cleaved perpendicular to the c-axis to expose optically flat areas and were mounted to the sample stage using Ge-varnish for minimal strain.

### Optical rotation and MOKE

**Fig. 2:**
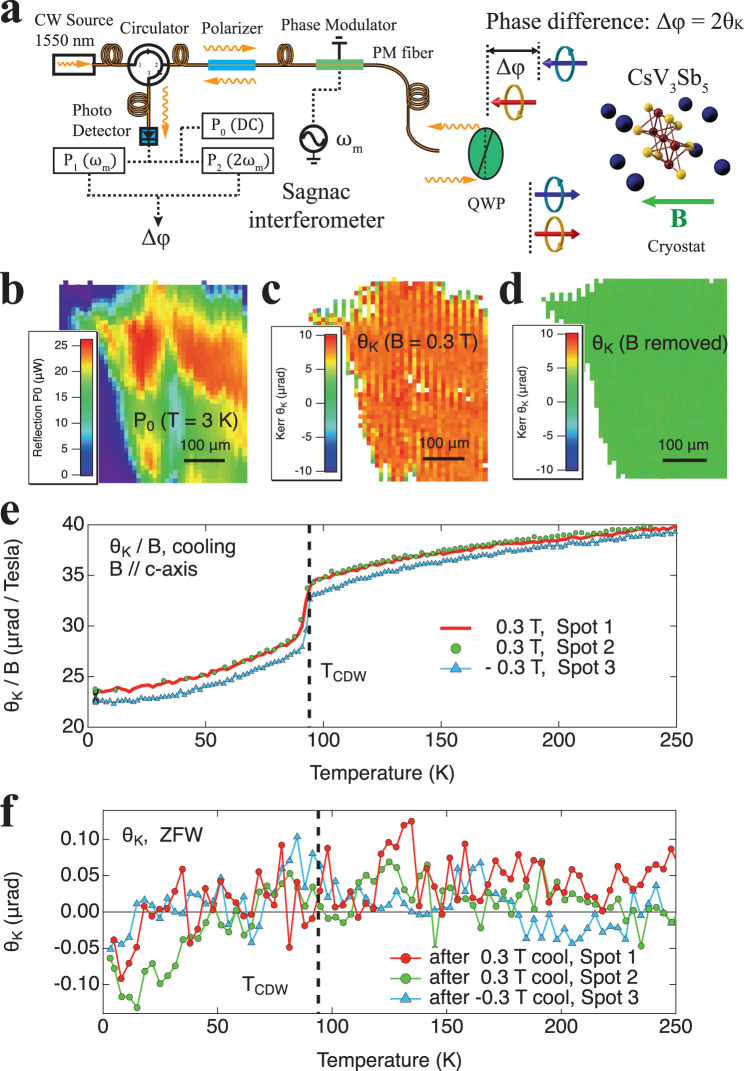
MOKE *θ*_*K*_ in CsV3Sb5 sample 1. **a** MOKE signal *θ*_*K*_ is measured by a Sagnac interferometer during the same experiment. See SI for details. **b**, **c** Scanning images of reflected optical power *P*_0_ and MOKE *θ*_*K*_ at 3 *K* in *B* = 0.3 *T* showing uniform *θ*_*K*_ in the reflective regions. **d** Image of *θ*_*K*_ after removal of the magnetic field, showing zero spontaneous MOKE signal across the sample. **e** *θ*_*K*_/*B* during cooldown at a few locations and magnetic fields showing a paramagnetic MOKE response that drops sharply below *T*_*CDW*_. **f** No onset of spontaneous *θ*_*K*_ at *T*_*CDW*_ was found during subsequent zero field warmup (ZFW).

As shown in Fig. 1a, a standard optical setup based on a Wollaston prism is used to measure the optical rotation (*θ*_*T*_) as a function of incident polarization angle *α*. And a zero-loop Sagnac interferometer^32^ as shown in Fig. 2a is used for polar MOKE (*θ*_*K*_) detection and imaging. Both instruments are connected to the same optical cryostat so *θ*_*T*_ and *θ*_*K*_ can be obtained from the same region in the sample during one experiment. Operation and calibration of both instruments on several test samples are described in the Supplementary Information. As shown in Fig. S2, the resolution of *θ*_*T*_ and *θ*_*K*_ are 30 μrad and 0.02 μrad, respectively. And they agree within 2% on the MOKE signal of a magnetic film test sample. The root cause for the Sagnac interferometer’s superior sensitivity is that it only measures microscopic TRSB and rejects any non-TRSB effects such as anisotropy. This is because the sourcing aperture for one light is the receiving aperture for the other time-reversed counterpropagating light, both apertures being the same single mode optical fiber. Hence Onsager’s relations guarantee zero signal in the absence of microscopic TRSB. Such rejection of non-TRSB effect is demonstrated in Fig. S2e to the 0.04 μrad level with an anisotropic polymer film, which displays anisotropic polarization rotations with twofold rotational symmetry of ±20 mrad.

We first examine CsV_3_Sb_5_ sample 1, whose optical rotation and MOKE results are summarized in Figs. 1, 2, respectively. At *B* = 0, polar plots of *θ*_*T*_ (*α*) at representative temperatures are shown in Fig. 1b, with additional temperatures plotted in Fig. S3a-l. Below *T*_*CDW*_, the total rotation *θ*_*T*_ contains an anisotropic component *θ*_*P*_ and an isotropic component *θ*_*C*_, similar to the findings at the 800 nm wavelength^23,24^. Fitted values of *θ*_*P*_ and *θ*_*C*_ are plotted in Fig. 1d showing sharp onsets just below *T*_*CDW*_. The *θ*_*P*_ component originates from the reduction from sixfold rotational symmetry to 2-fold in the CDW state. Since electronic nematicity was found^35,36^ in CsV_3_Sb_5_ at much lower temperatures, *θ*_*P*_ is likely of structural origin. The size of *θ*_*P*_ is 0.2 *mrad* in our 1550 nm measurement. It should scale linearly with the anisotropies in the permittivity and magneto-electric tensors^37^ and inversely with the wavelength *λ*. The rather flat near-infrared spectra^33,34^ of CsV_3_Sb_5_ suggest that the former factor is comparable between 800 nm and 1550 nm. Hence, *θ*_*P*_ is expected to roughly double at 800 nm reaching 0.4 mrad, which indeed falls between the experimentally reported values of 0.9 mrad^24^ and 0.2 mrad^23^ at 800 nm.

What comes as a surprise is the size of the isotropic component *θ*_*C*_, which has been interpreted as MOKE (*θ*_*K*_) in the 800 nm experiments^23,24^ and is expected to be vanishingly small at 1550 nm. Instead, the observed *θ*_*C*_ as plotted in Fig. 1(d) reaches 1.4 *mrad*, which is *larger* than the reported values^23,24^ at 800 nm. A MOKE signal, which arises from TRSB, should flip sign with opposite magnetic fields. In contrast, we found no such response in *θ*_*C*_: as shown in Fig. 1(c) with ±0.3 *T* applied magnetic fields, the change of optical rotation ∆*θ*_*T*_ is much smaller than *θ*_*C*_, approaching the noise level. This field-insensitivity is also opposite to the TRSB signatures reported in both STM^15^ and µSR^16–18^ experiments, which show clear magnetic responses. All these observations are suggestive that the isotropic component *θ*_*C*_ reported in^23,24^ and in this work is not MOKE and is not related to TRSB.

The decisive evidence that *θ*_*C*_ is not MOKE comes from Sagnac measurements of the same sample. As explained earlier, a Sagnac interferometer is only sensitive to MOKE, which is a direct result of TRSB. Figure 2c, d is *θ*_*K*_ images of the same region in sample 1 at 3 *K* with *B* = 0.3 *T* and *B* = 0, respectively. In the optically flat region where the reflected optical power (Fig. 2(b)) *P*_0_ > 1 *μW*, the MOKE signal is uniform with *θ*_*K*_ ~ 7 μrad in *B* = 0.3 *T*, and vanishes after the field is removed. *θ*_*K*_ is found to be paramagnetic and the MOKE susceptibility *θ*_*K*_/*B* remains field-independent during cooldowns as shown in Fig. 2e. In another study^31^ we verified this paramagnetic MOKE response at fields up to 9 *T*. The absence of a Curie–Weiss shape in *θ*_*K*_/*B* leads us to attribute it to the Pauli paramagnetism. And the sharp drop in *θ*_*K*_/*B* from 32 *μrad*/*T* to 27 *μrad*/*T* at *T*_*CDW*_ is likely due to a decreased density of states in the CDW phase, which agrees with the reported reduction of magnetic susceptibility^3^. The magnetic fields were removed at the base temperature, and the subsequent zero field warmups (ZFW) are plotted in Fig. 2f. We observe no onset of spontaneous *θ*_*K*_ at *T*_*CDW*_ with an uncertainty of 0.02 μrad, which is five orders of magnitude smaller than the observed *θ*_*C*_ ~1.4 mrad in the same sample.

**Fig. 3:**
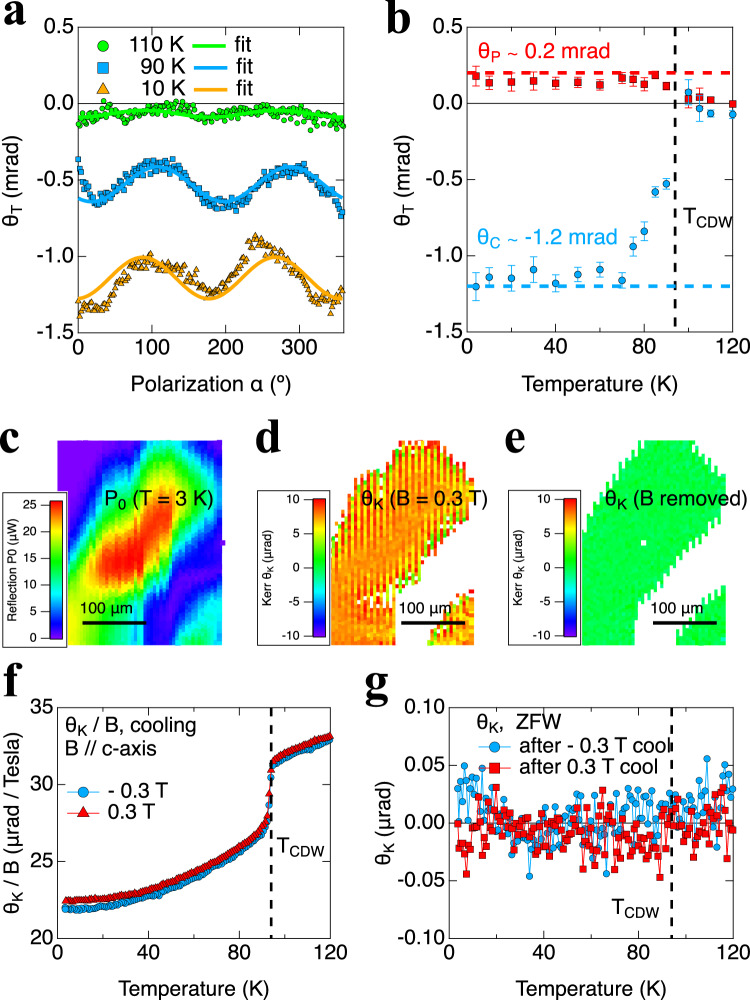
Optical rotation and MOKE in another CsV3Sb5 sample 2 with negative *θ*_*C*_. **a** Spontaneous *θ*_*T*_ at representative temperatures, fitted with *θ*_*T*_ = *θ*_*C*_ + *θ*_*P*_ sin(2*α* − *A*). See Fig. S4a–o for full data. **b** Fitted *θ*_*C*_ (*θ*_*P*_) up to −1.2 mrad (0.2 mrad) with onsets below *T*_*CDW*_, where error bars represent uncertainty in fitting. Note the negative sign of *θ*_*C*_. **c**–**e** are scanning images of *P*_0_, *θ*_*K*_ with *B* = 0.3 *T*, and *θ*_*K*_ after field removal, all at 3 *K*. **f**, **g** are averaged traces of *θ*_*K*_/*B* during field cooldowns and of spontaneous *θ*_*K*_ during subsequent ZFW. See SI for full data. There is no observable onset of spontaneous *θ*_*K*_ at *T*_*CDW*_ with an uncertainty of 0.02 μrad.

The sign of *θ*_*C*_ is also unrelated to that of *θ*_*K*_, and appears to have already been determined during crystal growth. On the same crystal, we have always found the same sign of *θ*_*C*_, regardless of direction of applied magnetic fields. The sign of *θ*_*C*_ can flip between samples. One such example can be found in CsV_3_Sb_5_ sample 2. The polarization rotations *θ*_*T*_(*α*) of sample 2 are mostly negative, as shown for representative temperatures in Fig. 3a, and for additional temperatures in Fig. S4a–o. The fitted anisotropic component *θ*_*P*_ and isotropic component *θ*_*C*_ are plotted in Fig. 3b, both showing sharp onsets at *T*_*CDW*_. The low-temperature value of *θ*_*P*_ (0.2 *mrad*) is comparable to that in sample 1, while *θ*_*C*_ ~ −1.2 mrad is similar in size but opposite in sign to that found in sample 1. The size of *θ*_*C*_ is also comparable to the 0.5 *mrad* value reported in a 800 nm experiment^24^ where *θ*_*C*_ of the same sign and similar sizes were found in two CsV_3_Sb_5_ samples. Interestingly in another 800 nm experiment^23^, *θ*_*C*_ of different signs and sizes up to 0.05 *mrad* were observed at different locations in a CsV_3_Sb_5_ sample. This can be explained if multi-domains of “sample 1” type and “sample 2” type are present in their sample, which will lead to location-dependent values of ±*θ*_*C*_, and even intermediate values for sub-wavelength (1 μm) domains. It may also explain why their measured *θ*_*C*_^23^ is one order of magnitude smaller than possible “single domain” samples in this work and in ref. ^24^. The sign of *θ*_*C*_ remains unchanged after thermal cycles in both samples 1 and 2, which agrees with the findings in the above 800 nm experiment^23^ that thermal cycles at the same location do not change *θ*_*C*_. In contrast, the MOKE signals *θ*_*K*_ (Fig. 3f) flip sign with opposite fields and are identical to those measured in sample 1. At zero magnetic field the change of spontaneous *θ*_*K*_ across *T*_*CDW*_ is below 0.02 μrad (Fig. 3g), which also agrees with the findings in sample 1.

## Discussion

With these observations, we conclude that the isotropic polarization component *θ*_*C*_ ~ ±(1.3 ± 0.1) *mrad* is not MOKE, which is five orders of magnitude smaller. And the sign of *θ*_*C*_, unlike the MOKE signal *θ*_*K*_, is unrelated to the direction of applied magnetic fields, but seems to have been predetermined at crystal growth. Therefore, *θ*_*C*_ is not related to the proposed TRSB loop currents^19,20^ or experimentally observed TRSB signatures in both STM^15^ and µSR^16–18^ experiments. Being isotropic, *θ*_*C*_ also does not originate from anisotropy, which gives rise to the anisotropic component *θ*_*P*_ ~ 0.2 mrad.

The expected MOKE signal estimated from the sub-Gauss level internal magnetic field measured in µSR^16–18^ experiments is at the nanoradian to sub-microradian level^27,28^ if the loop currents^19,20^ order ferromagnetically between adjacent layers. Since antiferromagnetic interlayer ordering may be more favorable energetically, the expected MOKE signal from TRSB may be much smaller, falling below our sensitivity of 0.02 μrad. The TRSB signature can still be detected by STM^15^ that probes the top layer, and µSR^16–18^ that senses local fields. Optical resonance enhancements would likely be needed for Sagnac MOKE to detect a TRSB signal that would require the operation in the Terahertz, which hasn’t been developed yet.

The observed *θ*_*C*_, an isotropic specular optical rotation at normal incidence without MOKE, represents new physics beyond TRSB or any other order discussed in AV_3_Sb_5_. The very existence of this unconventional optical effect, often referred to as specular (reflection) optical rotation^38^, is a matter of long-standing debate and is usually rejected^25^ in the context of ordinary optical materials. It was theoretically proposed to exist in strongly absorbing nematic materials and was reported in α-HgS (cinnabar) at 543 nm, close to the bandgap energy^38^. However, neither the 1550 nm or the 800 nm wavelength is close to a band gap in CsV_3_Sb_5_^33,34^. In addition, it has been shown^39^ that a nematic material such as cinnabar would not produce such isotropic rotation. Instead^39^, for nematic materials with optical activity the rotation is proportional to the difference of the magneto-electric tensor^37^ components *k*_*xx*_ − *k*_*yy*_ perpendicular to the propagation direction *z*, which is the c-axis of CsV_3_Sb_5_. As such, the resulting optical rotation would flip sign when the incidence polarization angle *α* is rotated by 90°, which is incompatible with the isotropic nature of *θ*_*C*_. In fact, this nematic optical activity could produce an optical rotation ∝(*k*_*xx*_ − *k*_*yy*_) cos(2*α*) (see discussions in the Supplementary Information) that would explain the puzzling sinusoidal form of the anisotropic rotation component *θ*_*P*_ sin(2*α* − *A*).

Hence the observed isotropic *θ*_*C*_ does not originate from a chiral order such as a cholesteric CDW state but is the first clear demonstration of the long-sought isotropic specular optical rotation not found in any other material system to our knowledge. And this isotropic rotation *θ*_*C*_ observed at 800 nm^23,24^ and at 1550 nm in this work either indicates a new intertwined order, or represents a novel hybrid phenomenon from the complex intertwining of the various orders in the CDW state. The topological Kagome lattice^40^ is indeed full of surprises.

## Methods

High-quality CsV_3_Sb_5_ single crystals were grown by the self-flux method at UCSB. Heat capacity and electrical transport measurements were performed with a Quantum Design Physical Property Measurement System (PPMS) at UC Irvine. Both polarization rotation and MOKE measurements were performed in an optical cryostat without breaking vacuum at UC Irvine, where polarization rotation is measured with the standard Wollaston prism method, and MOKE is conducted with a fiber-optics Sagnac interferometer microscope. Both optical instruments’ working principles are briefly described below, and their operation, performance, and cross-checking are detailed in the Supplementary Information. They operate with continuous wave (CW) light sources at the 1550 nm wavelength, with typical optical powers of 100 μW in the polarization rotation setup and 20 μW in the Sagnac interferometer.

The polarization rotation setup is shown in Fig. 1(a). The beam of light is routed through a free-space polarizer to produce a linearly polarized beam. A polarization-independent beam splitter (half mirror) transmits half of the beam and reflects the other half, which is discarded. The transmitted beam passes through a pinhole and then a half-wave plate (HWP), which is mechanically rotated such that its principal fast axis is at an angle *α*/2 to the polarization direction of the beam. The resulting beam after the HWP has its polarization direction rotated by angle *α*. The beam then passes through the optical window of the cryostat and gets reflected by the sample. The returned light beam passing the same pinhole a second time is in general elliptical with the major axis rotated by the total polarization rotation *α* +*θ*_*T*_. After passing through the HWP a second time, its polarization direction is rotated by −*α*, and becomes *θ*_*T*_. And the same polarization-independent beam splitter reflects half the returned beam towards a Wollaston prism, which separates and directs two orthogonal polarizations s and p toward two balanced detectors. The recorded powers of s and p-polarization components are P1 and P2 respectively. The Wollaston prism is rotated at a *π*/4 angle such that with a gold mirror calibration sample P1 and P2 are “balanced”: ∆*P* = *P*1 − *P*2 ~ 0. The optical amplitudes *E*1 and *E*2 at detectors 1 and 2 are:

1 $$E1=E0\,{{\cos }}\left(\frac{\pi }{4}-{\theta }_{T}\right)$$

2 $$E2=E0\,{{\cos }}\left(\frac{\pi }{4}+{\theta }_{T}\right)$$

where *E*0 is the total amplitude. Since optical intensity *I* = *E*^2^, the sum and difference of the two intensities *I*1 and *I*2 are:

3 $$\,I1+\,I2={E1}^{2}+{E2}^{2}=\,{E0}^{2}{{{\cos }}}^{2}\left(\frac{\pi }{4}+{\theta }_{T}\right) \\+{E0}^{2}{{{\cos }}}^{2}\left(\frac{\pi }{4}-{\theta }_{T}\right)={E0}^{2}$$

4 $$I1-\,I2=	 \,{E1}^{2}-{E2}^{2}=\,{E0}^{2}{{{\cos }}}^{2}\left(\frac{\pi }{4}-{\theta }_{T}\right)-{E0}^{2}{{{\cos }}}^{2}\left(\frac{\pi }{4}+{\theta }_{T}\right) \\=	 {E0}^{2}{{\sin }}(2{\theta }_{T})$$

Hence:

5 $$\frac{I1-\,I2}{I1+\,I2}=\,{{\sin }}(2{\theta }_{T})$$

As optical power is proportional to intensity *P* ∝ *I*, we can extract *θ*_*T*_ as:

6 $${\theta }_{T}\,=\frac{1}{2}{{\arcsin }}\left(\,\frac{I1-\,I2}{I1+\,I2}\right)=\,\frac{1}{2}{{\arcsin }}\left(\,\frac{P1-\,P2}{P1+\,P2}\right)=\,\frac{1}{2}{{\arcsin }}\left(\,\frac{\Delta P}{P1+\,P2}\right)$$

The MOKE measurement is performed using a zero-loop fiber-optic Sagnac interferometer as shown in Fig. 2a. The beam of light is routed by a fiber circulator to a fiber polarizer. After the polarizer the polarization of the beam is at 45° to the axis of a fiber-coupled electro-optic modulator (EOM), which generates 4.6 MHz time-varying phase shifts *ϕ*_*m*_ sin(*ωt*), where the amplitude *ϕ*_*m*_ = 0.92 rad between the two orthogonal polarizations that are then launched into the fast and slow axes of a polarization maintaining (PM) single-mode fiber. Upon exiting the fiber, the two orthogonally polarized linearly polarized beams are converted into right- and left-circularly polarizations by a quarter-wave plate (QWP) and are then focused onto the sample. After reflection from the sample, the same QWP converts the reflected beams back into linear polarizations with exchanged polarization axes. The two beams then pass through the PM fiber and EOM but with exchanged polarization modes in the fiber and the EOM. At this point, the two beams have gone through the same path but in opposite directions, except for a phase difference of ∆*φ* from reflection off the magnetic sample and another time-varying phase difference by the modulation of EOM. This nonreciprocal phase shift ∆*φ* between the two counterpropagating circularly polarized beams upon reflection from the sample is twice the Kerr rotation ∆*φ* = 2*θ*_*K*_. The two beams are once again combined at the detector and interfere to produce an optical signal *P*(*t*):

7 $$P\left(t\right)=\,\frac{1}{2}\,P[1+{{\cos }}(\Delta \varphi+{\phi }_{m}\,{{\sin }}(\omega t))]$$

where *P* is the returned power if the modulation by the EOM is turned off. For MOKE signals that are slower that the 4.590 MHz modulation frequency used in this experiment, we can treat ∆*φ* as a slowly time-varying quantity. And *P*(*t*) can be further expanded into Fourier series with the first few orders listed below:

8 $$P\left(t\right)/P=	 \frac{1}{2}\,[1+{{{{{{\rm{J}}}}}}}_{0}({2\phi }_{m})]\\ 	+({{\sin }}\left(\Delta \varphi \right){{{{{{\rm{J}}}}}}}_{1}({2\phi }_{m}))\,{{\sin }}(\omega t),\\ 	+({{\cos }}\left(\Delta \varphi \right){{{{{{\rm{J}}}}}}}_{2}({2\phi }_{m}))\,{{\cos }}(2\omega t)\\ 	+2\,{{{{{{\rm{J}}}}}}}_{3}({2\phi }_{m})\left)\right.\,{{\sin }}(3\omega t)\,+\ldots$$

where *J*_1_(2*ϕ*_*m*_) and *J*_2_(2*ϕ*_*m*_) are Bessel J-functions. Lock-in detection was used to measure the first three Fourier components: the average (DC) power (P0), the first harmonics (P1), and the second harmonics (P2). And the Kerr rotation can then be extracted using the following formula:

9 $${\theta }_{K}=\frac{1}{2}\,\Delta \varphi=\frac{1}{2}{\tan }^{-1}\left[\frac{{J}_{2}\left(2{\phi }_{m}\right)P1}{{J}_{1}\left(2{\phi }_{m}\right)P2}\right]$$

## Reporting summary

Further information on research design is available in the Nature Portfolio Reporting Summary linked to this article.

### Supplementary information

Supplementary Information

Peer Review File

## Data Availability

Source data are provided with this paper. They have been deposited in a figshare repository with 10.6084/m9.figshare.23575155.
