# Supplementary material for: Unconventional specular optical rotation in the charge ordered state of Kagome metal CsV3Sb5
Source: Nat Commun. 2023 Sep 1;14:5326. doi: 10.1038/s41467-023-41080-5 (PMC10474032; doi:10.1038/s41467-023-41080-5)
Supplement: Supplementary file 1 — Supplementary Information [file 41467_2023_41080_MOESM1_ESM.pdf]

Supplementary Information for

Unconventional Specular Optical Rotation in the Charge Ordered State of Kagome Metal CsV_3Sb_5

Camron Farhang[1], Jingyuan Wang[1], Brenden R. Ortiz[2], Stephen D. Wilson[2] and Jing Xia[1]

[1]*Department of Physics and Astronomy, University of California, Irvine, California 92697, USA*

[2]*Materials Department, University of California, Santa Barbara, Santa Barbara, CA 93106, USA.11973*

(A) Methods for measuring polarization rotation and MOKE

To resolve the controversy on the optical rotation of CsV_3Sb_5, it is important to measure both polarization rotation θ_T and MOKE θ_K on the same sample using the same optical wavelength and under the same experimental conditions. To achieve this, we have constructed a standard Wollaston-prism-based polarization rotation setup that can be connected to the same optical cryostat for the Sagnac interferometer microscope. This configuration allows both types of measurements on the same crystal (usually in the same region) without leaving the vacuum of the cryostat. Both instruments operate with continuous wave (CW) light sources at the 1550 nm wavelength. The typical optical powers used in the experiments are 100 μW in the polarization rotation setup and 20 μW in the Sagnac interferometer. This amount of optical power has negligible heating effects at $T_{CDW} \sim 94\ K$. Normal incidence reflection is ensured in the polarization setup with a pinhole, and in the Sagnac interferometer with a single mode fiber.

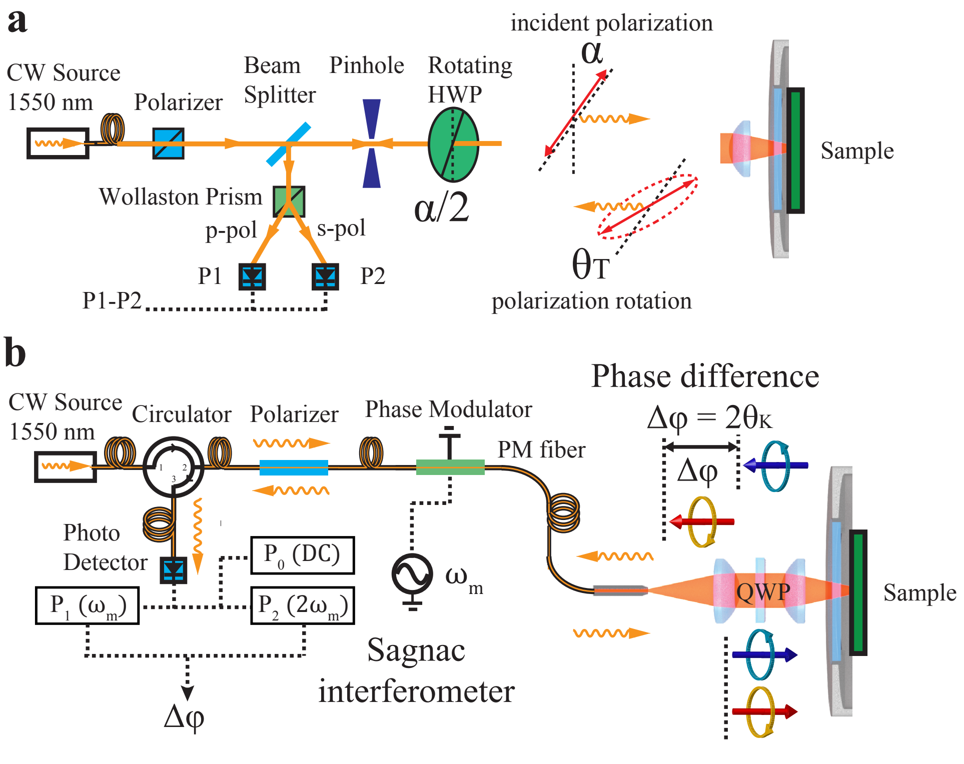

Fig.S1. Polarization rotation and MOKE setups operating with continuous-wave (CW) light at 1550 nm wavelength: *Both optical setups are connected to the same optical cryostat to allow measurements on the same sample during one experiment.* ***(a)*** *Schematics of polarization rotation setup based on a Wollaston prism that measures polarization rotation θ_T as a function of incident polarization α, which is achieved by rotating a half-wave plate (HWP) by $\alpha/2$.* ***(b)*** *Schematics of a zero-area-loop fiber-optic interferometer that is only sensitive to TRSB (MOKE θ_K) effects, which is independent of α. The fiber-optic head can be scanned to simultaneously acquire reflection and MOKE images.*

The schematics of the polarization rotation setup is shown in Fig.S1(a). The beam of light from a CW light source centered at 1550 nm is routed through a free-space polarizer to produce a linearly polarized beam. A polarization-independent beam splitter (half mirror) transmits half of the beam and reflects the other half, which is discarded. The transmitted beam passes through a pinhole and then a half-wave plate (HWP), which is mechanically rotated such that its principle fast axis is at an angle $\alpha/2$ to the polarization direction of the beam. The resulting beam after the HWP has its polarization direction rotated by angle α. The purpose is to allow the control of the relative angle α between the incident polarization and the same crystal axis without rotating the sample itself, which is difficult to do in the cryostat and will introduce noise and offsets. The beam then passes through the optical window of the cryostat and gets reflected by the sample. The returned light beam is in general elliptical with the major axis rotated by the total polarization rotation $\alpha + \theta_T$. After passing through the HWP a second time, its polarization direction is rotated by $-\alpha$, and becomes θ_T. The optical path is aligned such that the beam can pass the same pinhole a second time, ensuring normal incidence reflection from the sample. Then the same polarization-independent beam splitter will reflect half the returned beam towards a Wollaston prism, which separates and directs two orthogonal polarizations s and p towards two balanced detectors. The recorded powers of s and p-polarization components are P1 and P2 respectively. The Wollaston prism is rotated at a $\pi/4$ angle such that with a gold mirror calibration sample ($\theta_T = 0$), P1 and P2 are "balanced": $\Delta P = P1 - P2 \sim 0$. In this configuration, one can show that the optical amplitudes $E1$ and $E2$ at detectors 1 and 2 are:

$$E1 = E0 \cos\left(\frac{\pi}{4} - \theta_T\right) \tag{1}$$

$$E2 = E0 \cos\left(\frac{\pi}{4} + \theta_T\right) \tag{2}$$

, where $E0$ is the total amplitude. Since optical intensity $I = E^2$, the sum and difference of the two intensities $I1$ and $I2$ are:

$$I1 + I2 = E1^2 + E2^2 = E0^2 \cos^2\left(\frac{\pi}{4} + \theta_T\right) + E0^2 \cos^2\left(\frac{\pi}{4} - \theta_T\right) = E0^2 \tag{3}$$

$$I1 - I2 = E1^2 - E2^2 = E0^2 \cos^2\left(\frac{\pi}{4} - \theta_T\right) - E0^2 \cos^2\left(\frac{\pi}{4} + \theta_T\right) = E0^2 \sin(2\theta_T) \tag{4}$$

Hence:

$$\frac{I1 - I2}{I1 + I2} = \sin(2\theta_T) \tag{5}$$

As optical power is proportional to intensity $P \propto I$, we can extract θ_T as:

$$\theta_T = \frac{1}{2}\arcsin\left(\frac{I1 - I2}{I1 + I2}\right) = \frac{1}{2}\arcsin\left(\frac{P1 - P2}{P1 + P2}\right) = \frac{1}{2}\arcsin\left(\frac{\Delta P}{P1 + P2}\right) \tag{6}$$

, where ΔP is read from a direct output of the balanced detector. Optical components such as the focusing lens and cryostat optical window have μrad to $mrad$ levels of optical birefringence due to residual strains. Fortunately, these contributions are independent of sample temperature since these optical components are outside of the cryostat and are at the fixed room temperature. In this work, a base line value at 120 K, $\theta_T(120\ K)$ is subtracted from θ_T to remove this background.

The schematics of the zero-loop Sagnac interferometer used in this work is shown in Fig.S1(b). The beam of light from a CW light source centered at 1550 nm is routed by a fiber-circulator to a fiber-polarizer, which polarizes the beam. The circulator transmits light from port 1 to port 2 and from port 2 to port 3 with better than 30 dB isolation in the reverse directions. After the polarizer the polarization of the beam is at 45° to the axis of a fiber-coupled electro-optic modulator (EOM), which generates 4.6 MHz time-varying phase shifts $\phi_m \sin(\omega t)$, where the amplitude $\phi_m = 0.92$ rad between the two orthogonal polarizations that are then launched into the fast and slow axes of a polarization maintaining (PM) single mode fiber. Upon exiting the fiber, the two orthogonally polarized linearly polarized beams are converted into right- and left-circularly polarizations by a quarter-wave plate (QWP) and are then focused through the optical window of the cryostat onto the sample. After reflection from the sample and passing through the optical window, the same quarter-wave plate converts the reflected beams back into linear polarization with exchanged polarization axes. The two beams then pass through the PM fiber and EOM but with exchanged polarization modes in the fiber and the EOM. At this point, the two beams have gone through the same path but in opposite directions, except for a phase difference of $\Delta\varphi$ from reflection off the magnetic sample and another time-varying phase difference by the modulation of EOM. This nonreciprocal phase shift $\Delta\varphi$ between the two counterpropagating circularly polarized beams upon reflection from the sample is twice the Kerr rotation $\Delta\varphi = 2\theta_K$. The two beams are once again combined at the detector and interfere to produce an optical signal $P(t)$:

$$P(t) = \frac{1}{2} P[1 + \cos(\Delta\varphi + \phi_m \sin(\omega t))] \tag{7}$$

, where P is the returned power if the modulation by the EOM is turned off. For MOKE signals that are slower that the 4.590 MHz modulation frequency used in this experiment, we can treat $\Delta\varphi$ as a slowly time-varying quantity. And $P(t)$ can be further expanded into Fourier series with the first few orders listed below:

$$\begin{aligned} P(t)/P = & \frac{1}{2}\left[1 + \mathrm{J}_0(2\phi_m)\right] \\ & +(\sin(\Delta\varphi)\,\mathrm{J}_1(2\phi_m))\sin(\omega t) \\ & +(\cos(\Delta\varphi)\,\mathrm{J}_2(2\phi_m))\cos(2\omega t) \\ & +2\,\mathrm{J}_3(2\phi_m))\sin(3\omega t) \\ & +\cdots \end{aligned} \quad (8)$$

, where $J_1(2\phi_m)$ and $J_2(2\phi_m)$ are Bessel J-functions. Lock-in detection was used to measure the first three Fourier components: the average (DC) power (P0), the first harmonics (P1), and the second harmonics (P2). And the Kerr rotation can then be extracted using the following formula:

$$\theta_K = \frac{1}{2}\,\Delta\varphi = \frac{1}{2}\tan^{-1}\left[\frac{J_2(2\phi_m)P1}{J_1(2\phi_m)P2}\right] \quad (9)$$

The noise in Kerr signal is shot-noise-limited to $10^{-7}rad/\sqrt{Hz}$ with $10\ \mu W$ of optical power, which is small enough not to heat up the sample even at the base temperature of the cryostat. By averaging over 100 seconds, 10 nanoradian (nrad) Kerr resolution can be achieved over a few Kelvins variation of sample temperatures. In practice, the bias offset in our system drifts about 20 nrad in experiments that take a long time or over wide sample temperature ranges. And the flexible fiber head can be mechanically scanned to simultaneously produce reflection (P0) and MOKE (θ_K) images.

(B) Cross-checking both optical setups with test samples

We have used a few test samples to cross-check the polarization rotation setup and the Sagnac interferometer. They are an uncoated gold mirror to evaluate offsets and noise, a magneto-optic thin film with a known MOKE signal to double check their calibrations, and a birefringent polyethylene polymer film to demonstrate the two setups different response to TRSB and non-TRSB optical rotations. Both total polarization rotation θ_T and the MOKE θ_K are measured as a function of the relative angle α between the incident polarization and the sample. With the polarization rotation setup, angle α is achieved by rotating the half-wave plate (HWP) by an angle $\alpha/2$. With the Sagnac interferometer, it is achieved by mounting the test samples on a rotational stage, which is rotated by angle α.

The results on the gold mirror are plotted in Fig.S2(a), with the polar plots shown in Fig.S1(b). The uncoated gold mirror should introduce near-zero optical rotations and thus serves as a null test sample. The MOKE θ_K readings (red squares) are zero with 10s of $nrad$ uncertainty, regardless of the sample angle α. This is the expected behavior for a Sagnac interferometer on a non-TRSB sample. The measured total polarization rotation θ_T (blue circles) scatter between 0 μrad and 30 μrad with most of the points falling near 20 μrad. The 20 μrad is the offset mostly likely due to slight misalignments of the Wollaston prism, which will be eliminated in temperature-dependent measurements of CsV_3Sb_5 by subtracting the base line value at 120 K sample temperature, $\theta_T(120\ K)$. The 30 μrad scattering represents the instrumentation noise due to various effects such as vibration and air flow and can be much larger in the CsV_3Sb_5 measurements due to the introduction of the cryostat. We note that these noise sources are non-TRSB and are rejected by the Sagnac interferometer.

To ensure that the scale calibrations of both instruments are correct, we measure a magneto-optic (MO) film (XP33BC32) that generates a MOKE signal of around 4 $mrad$ at the 1550 nm wavelength. Such a MOKE signal would present itself as an isotropic (polarization independent) rotation component $\theta_T(\alpha) = \theta_C$ in the polarization rotation measurement. A small magnetic field of 5 mT is applied to align the ferromagnetic domains in the MO film. The results and their polar plots are shown in Fig.S2(c) and Fig.S2(d). The measured polarization rotation $\theta_T(\alpha)$ (blue circles) vary between 4.38 $mrad$ and 4.43 $mrad$ between incident polarization angles α, and can be regarded as mostly isotropic, which is expected for a MOKE signal. The 50 μrad or 1% variance could arise from small linear birefringence in the film due to residual strains but could also be due to the instrumental noise and drift. The measured MOKE signal $\theta_K(\alpha)$ (red squares) vary between 4.35 $mrad$ and 4.47 $mrad$ between sample angles α. This 80 μrad (2 %) variance is much larger than the 0.02 μrad noise level of a Sagnac interferometer, whose reading should also not depend on the sample angles α. Since the sample is rotated mechanically in the Sagnac measurements, where the axis of rotation is not necessarily aligned with the optical beam, we suspect that the optical beam was probing different locations along a circular path at different sample angles α. And the inhomogeneity in the sample is the source of this 80 μrad variance. Nevertheless, this MO film demonstrates that the calibrations of the polarization setup and the Sagnac interferometer agree within 2 %, which is more than enough to compare the results from both instruments with sufficient accuracy and to reach the conclusions of this paper.

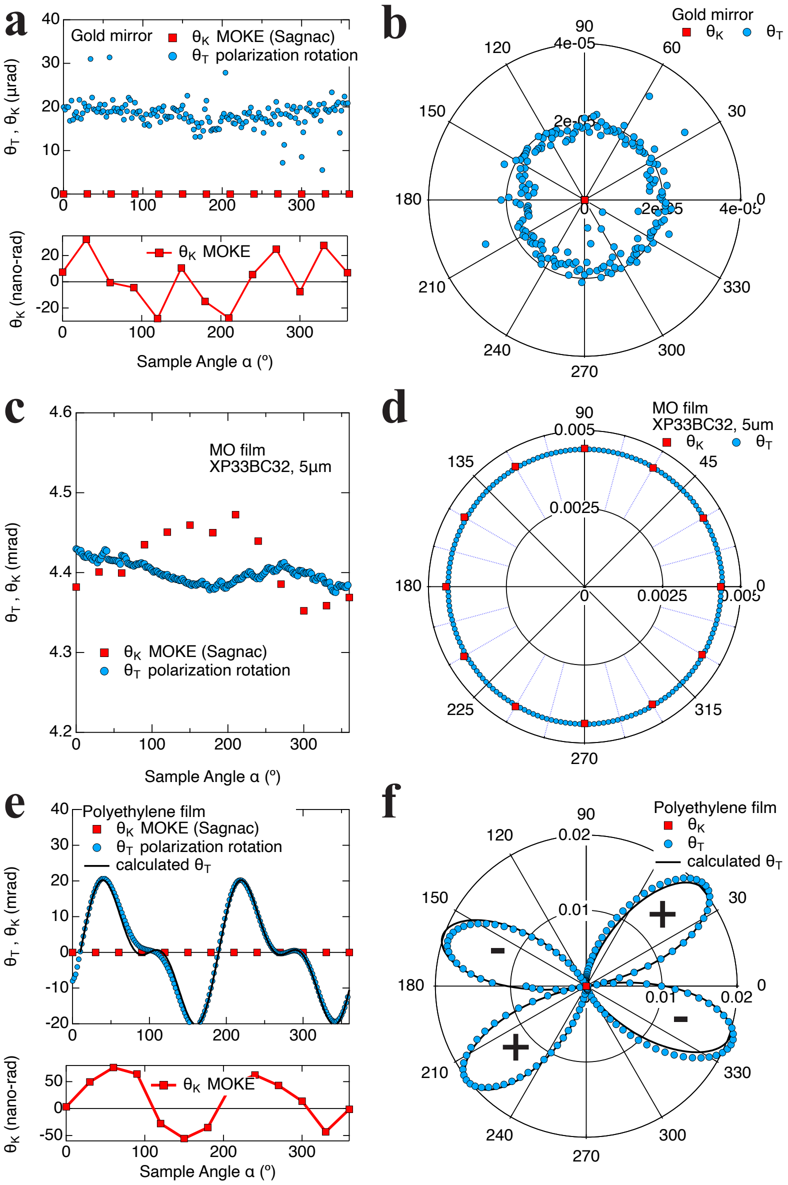

Fig.S2. Cross-checking polarization rotation θ_T and MOKE θ_K on test samples. *Sample angle α is changed by sample rotation with the Sagnac MOKE setup, and by rotating the half-wave plate by $\alpha/2$ in the polarization setup.* ***(a)(b)*** *An uncoated gold mirror with zero expected rotations. Measured θ_K is smaller than 30 nrad while θ_T has an instrument related 20 μrad offset.* ***(c)(d)*** *A magneto-optic film with ~ 4 mrad of MOKE signal. Measured θ_K and θ_T agree with each other within 2%.* ***(e)(f)*** *An anisotropic polyethylene film. Measured θ_K is smaller than 30 nrad as expected from a time-reversal symmetry invariant sample. θ_T shows a pattern that clearly demonstrates an anisotropic (rotational symmetry breaking) component θ_P, with zero isotropic (polarization-independent) rotation component (θ_C). Solid line is the calculated polarization rotation for an anisotropic reflective sample, and it agrees well with the measured θ_T.*

Finally, a polyethylene film is used to demonstrate the different responses to different polarization components between the two instruments. This polymer film is optically anisotropic and thus produces anisotropic optical rotations. The results and their polar plots are shown in Fig.S2(e) and Fig.S2(f). The measured MOKE signal $\theta_K(\alpha)$ (red squares) remains zero as the sample doesn't break time-reversal symmetry, while the measured polarization rotation $\theta_T(\alpha)$ (blue circles) up to $\pm 20\ mrad$ displays a four-leaf clover shape with 2-fold rotational symmetry. The shape is a direct result of the presence of optical linear birefringence (LB) and optical linear dichroism (LD). And it can be calculated analytically (see section F) and be

compared with the experimental curve to fit LB and LD parameters. In Fig.S2(e) and Fig.S2(f) I present the calculated $\theta_T(\alpha)$ curve (black line) using parameters LB = 0.133 and LD = 0.015, which matches well to the experimental data (blue circles). This polymer film serves as an example that while the polarization rotation setup detects the total rotation, the Sagnac interferometer is sensitive only to TRSB effects. We also note that in the reflection geometry, simple linearly birefringent and dichroic materials will often have a four-leaf clover shaped $\theta_T(\alpha)$ instead of a simple sinusoidal form $\theta_P\ sin(2\alpha - A)$ found in AV_3Sb_5.

(C) Full polarization rotation data of sample 1

In Fig.S3 we present polar plots of $\theta_T(\alpha)$ in CsV_3Sb_5 sample 1 at more temperatures. Fitted parameters of $\theta_C(T)$ and $\theta_P(T)$ are presented in the main text Fig.1(d).

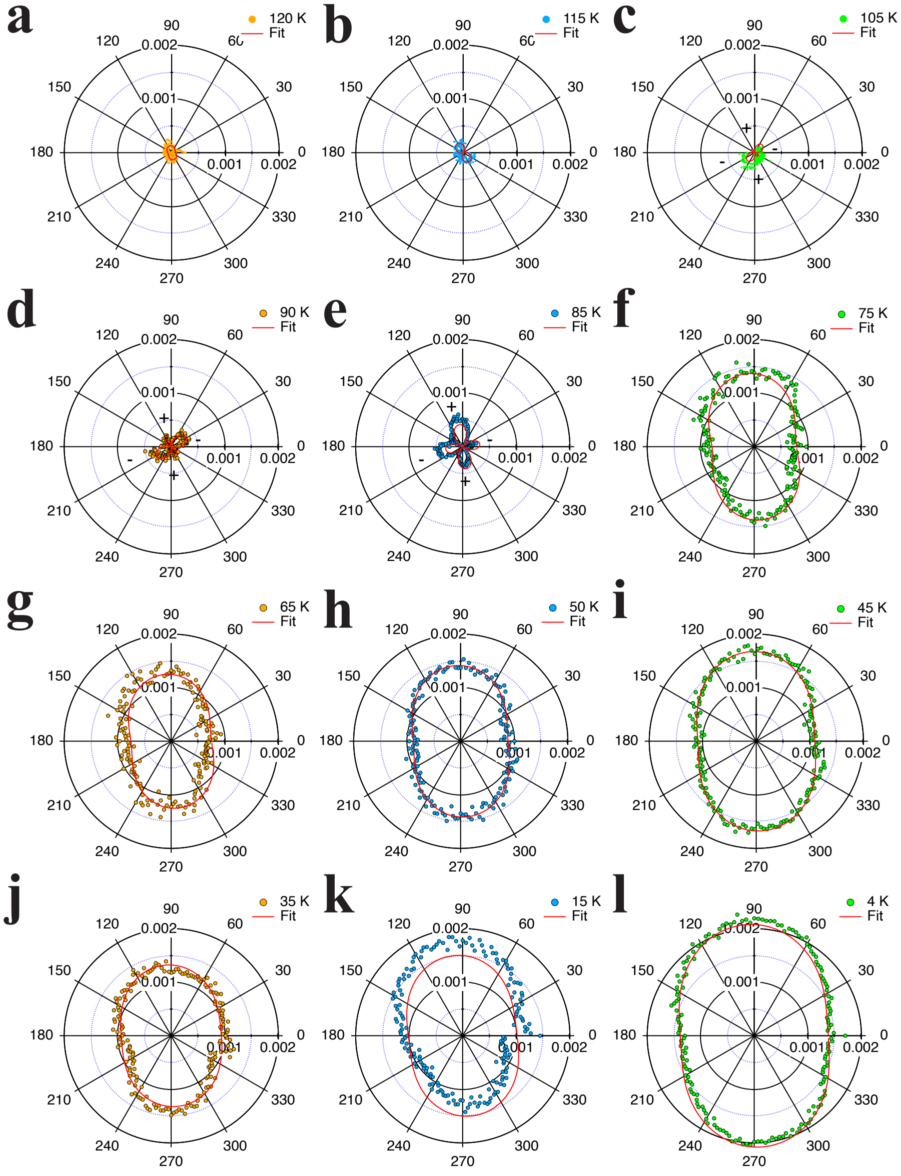

Fig.S3. Polarization rotations in sample 1: (a)-(l) *Polar plots of polarization rotations and fittings in sample 1 at various temperatures at zero magnetic field. The "-"sign indicates negative values.*

(D) Full polarization rotation data of sample 2

In Fig.S4 we present polar plots of $\theta_T(\alpha)$ in CsV_3Sb_5 sample 2. Fitted parameters of $\theta_C(T)$ and $\theta_P(T)$ are presented in the main text Fig.3(b).

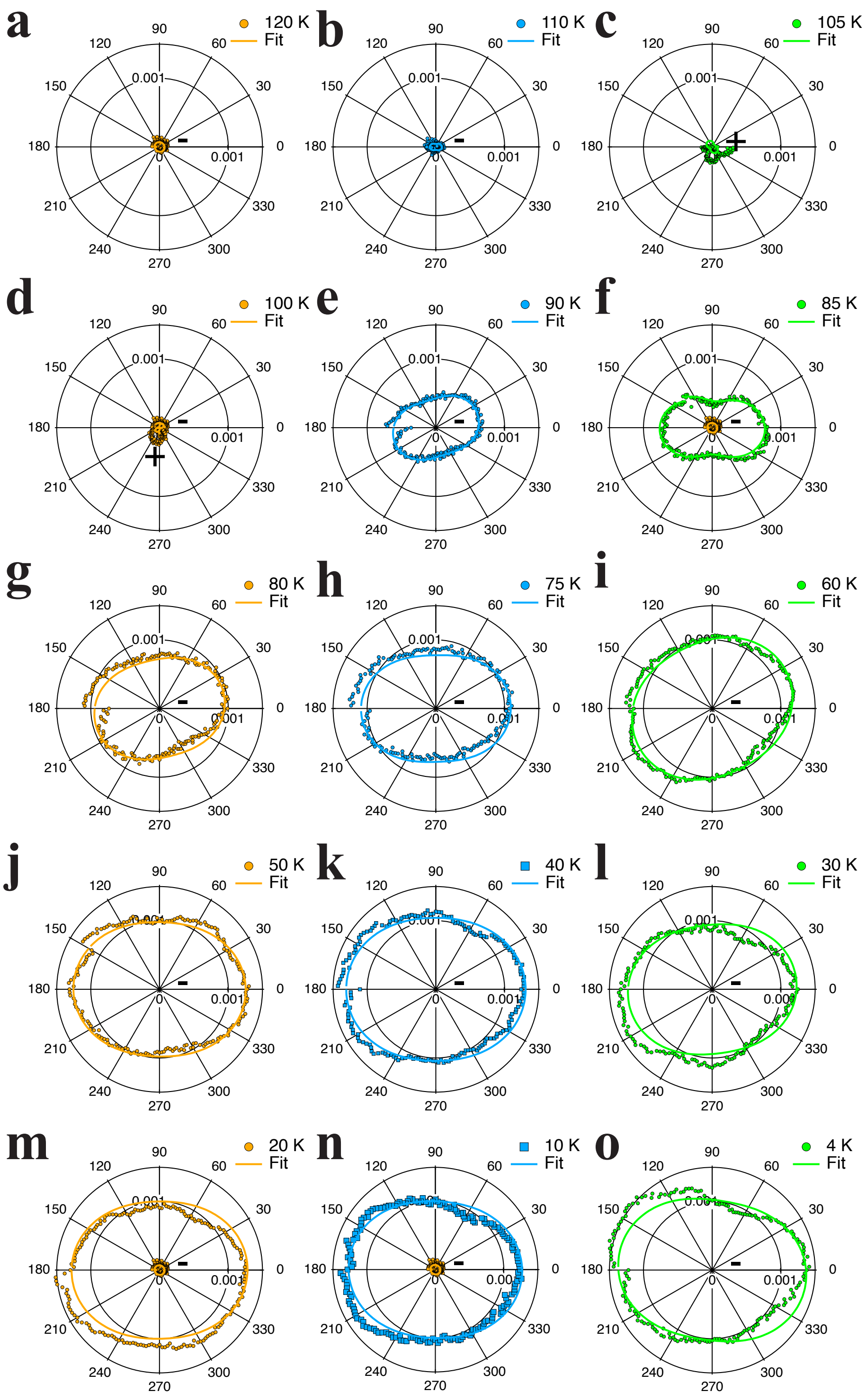

Fig.S4. Polarization rotations in sample 2: (a)-(o) *Polar plots of polarization rotations and fittings in sample 2 at various temperatures with zero magnetic field. The "-" sign indicates negative values.*

(E) Full MOKE data of sample 2

In Fig.S5 we present temperature traces of $\theta_K(T)/B$ in CsV_3Sb_5 sample 2 with a magnetic field, and temperature traces of $\theta_K(T)$ during zero field warmup (ZFW). 1 μrad/T or 1 μrad offsets are introduced for clarify. These traces are numerical averaged to generate traces that are presented in the main text Fig.3(f) and (g).

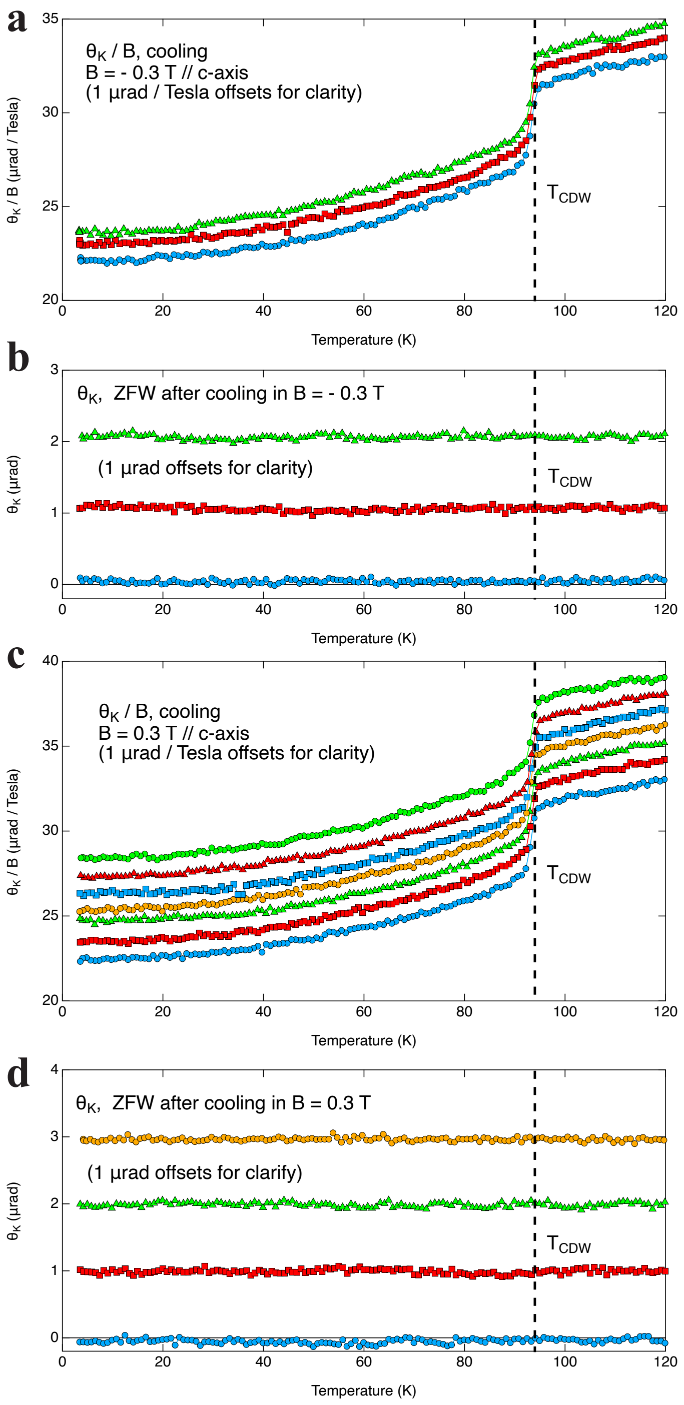

Fig.S5. MOKE data of sample 2: (a) *θ_K/B during cooldowns in -0.3 T magnetic field, with 1 $\mu rad/T$ offsets for clarity.* ***(b)*** *θ_K during subsequent zero-field warmups (ZFW), with 1 μrad offsets for clarity.* ***(c)*** *θ_K/B during cooldowns in 0.3 T magnetic field, with 1 $\mu rad/T$ offsets for clarity. (d) θ_K during subsequent zero-field warmups (ZFW), with 1 μrad offsets for clarity.*

(F) The origin of the observed anisotropic rotation component $\theta_P \, sin(2\alpha - A)$

The observed total polarization $\theta_T(\alpha)$ in both 800 nm reflection experiments and in this work (1550 nm) contains an anisotropic component in the form of $\theta_P \, sin(2\alpha - A)$, which has often been thought to be caused by optical linear anisotropy (linear birefringence and dichroism). However, using the Jones matrix, one can show that the polarization rotation of a sample with linear birefringence (LB) and linear dichroism (LD) has the following analytical form:

$$\theta_T(\alpha) = sin(2\,\alpha)cos(2\,\alpha)\,\frac{e^{i\,2\,LB}\,((LD-2)\,LD\,(2+(LD-2)\,LD)\,e^{i\,2\,LB} + (-(LD-1)^2 - e^{i\,4\,LB}\,(LD-1)^2 + e^{i\,2\,LB}(2+(LD-2)\,LD\,(2+(LD-2)\,LD)))}{2+(LD-2)\,LD\,(2+(LD-2)\,LD) + (-2+LD)\,LD\,(2+(LD-2)\,LD)\,cos(2\,\alpha))} \tag{10}$$

, which usually has the rather complicated four-leaf clover shape in Fig.S2(e) and Fig.S2(f). If linear dichroism LD is not present, the above formula is reduced to a simpler form:

$$\theta_T(\alpha) = sin^2(LB)\,sin(4\,\alpha) \tag{11}$$

It displays a 4-fold rotational symmetry pattern with sample angle α, which is still different from the observed 2-fold symmetric pattern $\theta_P \, sin(2\alpha - A)$ in CsV_3Sb_5 experiments. Therefore, the observed anisotropic θ_P component can't be explained by simple linear birefringence and/or dichroism that are often associated with a nematic CDW.

An alternative explanation is the reflection optical activity. As explained in *Opt. Lett.* **40**, 4277 (2015), at normal incidence reflection, such reflection optical activity is proportional to the difference of the magneto-electric tensor (*Rev. Mod. Phys.* **9**, 432–457 (1937)) components $k_{xx} - k_{yy}$ perpendicular to the propagation direction z, which is the c-axis of CsV_3Sb_5 in this work. Here the magneto-electric tensor describes the light-induced magnetization. As such, the resulting optical rotation would flip sign when the incidence polarization angle α is rotated by 90°, which is incompatible with the isotropic rotation component θ_C, but could explain the anisotropic rotation component $\theta_P sin(2\alpha - A)$ as the following. We consider the sub-tensor K_{2D} of the magneto-electric tensor in the xy-plane perpendicular to the incident light:

$$K_{2D} = \begin{bmatrix} k_{xx} & k_{xy} \\ -k_{xy} & k_{yy} \end{bmatrix} \tag{12}$$

Under a sample rotation α, this sub-tensor is transformed into:

$$K_{2D}(\alpha) = \begin{bmatrix} \frac{1}{2}(k_{xx} + k_{yy} + (k_{xx} - k_{yy})\cos(2\,\alpha)) & k_{xy} + \frac{1}{2}(k_{xx} - k_{yy})\sin(2\,\alpha) \\ -k_{xy} + \frac{1}{2}(k_{xx} - k_{yy})\sin(2\,\alpha) & \frac{1}{2}(k_{xx} + k_{yy} - (k_{xx} - k_{yy})\cos(2\,\alpha)) \end{bmatrix} \tag{13}$$

And the polarization rotation due to reflection optical activity is proportional to the difference between its diagonal components:

$$\theta_{OA}(\alpha) \propto K_{2D}(\alpha)_{xx} - K_{2D}(\alpha)_{yy} = (k_{xx} - k_{yy})\cos(2\,\alpha) \tag{14}$$

, which matches the observed sinusoidal form of the anisotropic rotation component $\theta_P sin(2\alpha - A)$, if angle A is set to $\pi/2$. Therefore, we propose that an anisotropic order in the CDW state generates a difference between k_{xx} and k_{yy} in the magneto-electric tensor, where xy plane is parallel to the cleaved surface perpendicular to the c-axis. It leads to an optical rotation component $\theta_P sin(2\alpha - A)$ that is observed in both 800 nm reflection experiments and in this work (1550 nm).
