# Supplementary material for: Unconventional specular optical rotation in the charge ordered state of Kagome metal CsV3Sb5
Source: Nat Commun. 2023 Sep 1;14:5326. doi: 10.1038/s41467-023-41080-5 (PMC10474032; doi:10.1038/s41467-023-41080-5)
Supplement: Supplementary file 2 — Peer Review File [file 41467_2023_41080_MOESM2_ESM.pdf]

nature portfolio

Peer Review File

Unconventional Specular Optical Rotation in the Charge Ordered State of Kagome Metal CsV3Sb5

Open Access This file is licensed under a Creative Commons Attribution 4.0 International License, which permits use, sharing, adaptation, distribution and reproduction in any medium or format, as long as you give appropriate credit to the original author(s) and the source, provide a link to the Creative Commons license, and indicate if changes were made. In the cases where the authors are anonymous, such as is the case for the reports of anonymous peer reviewers, author attribution should be to 'Anonymous Referee' followed by a clear attribution to the source work. The images or other third party material in this file are included in the article's Creative Commons license, unless indicated otherwise in a credit line to the material. If material is not included in the article's Creative Commons license and your intended use is not permitted by statutory regulation or exceeds the permitted use, you will need to obtain permission directly from the copyright holder. To view a copy of this license, visit http://creativecommons.org/licenses/by/4.0/.

Reviewers' Comments:

Reviewer #1:
Remarks to the Author:
The authors reported herein their optical rotation and polar MOKE measurements at a specific wavelength of 1550 nm on CsV_3Sb_5 crystals. They found a giant polarization independent isotropic components θ_c below the CDW temperature which do not respond to external magnetic field but flip the sign between samples. Meanwhile, the MOKE signal θ_k was found to be negligible. The authors argued that the observations indicate a weak link between the isotropic rotation and the experimentally observed time-reversal symmetry breaking by STM and other experiments. The authors attributed the isotropic rotation θ_c to either intertwined order or a novel hybrid phenomenon from the complex intertwining of the various orders in the CDW state, rather than time-reversal symmetry breaking, anisotropy or chiral order. This work not only clarified the discrepancy among various previous experiments reported different θ_c and θ_k and the relation between isotropic rotation and time-reversal symmetry breaking, but also clearly demonstrated the long-sought isotropic specular optical rotation in a solid.
This is an in-time and rigorous work that could stimulate much interest in further understanding the time-reversal symmetry breaking in these kagome compounds and the related intriguing physics. It may warrant its publication in Nature communications after addressing the following concerns:
1. The isotropic components θ_c show sample dependence, which in sample 2 are even opposite to that found in sample 1. The authors said "This can be explained if multi-domains of "sample 1" type and "sample 2" type are present in their sample, which will lead to location-dependent values of $\pm\theta_c$, and even intermediate values for sub-wavelength (1 μm) domains. It may also explain why their measured θ_c is one order of magnitude smaller than possible "single domain" samples in this work and in another 800 nm experiment where θ_c of the same sign and similar sizes were found in two CsV_3Sb_5 samples". It is somewhat astonishing that the different crystals can have so apparent difference in θ_c. If the difference is actually related to the domain sizes, it will be very crucial to characterize the domains of the crystals. Had the authors checked the domain sizes in their two crystals? A direct comparison of the domains of the two samples would be more straightforward for the readers to understand this argument.
2. On how many places of one crystal the authors measured the data? When measured different places of the same piece of crystal, are the results reported herein reproducible?
3. It is well known that loss of surface Cs tends to occur in CsV_3Sb_5, which can induce hole doping effect but could be very different in different samples. Will this bring influences on optical rotation and polar MOKE measurements?
4. After cleaved the crystals for measurements, how the authors guaranteed the crystal quality during measurements? Will the crystals be degenerated during measurements?
5. The time-reversal symmetry breaking is tightly related to the 3D CDW in CsV_3Sb_5 which is thickness dependent, where the change of CDW phase may have influence on the TRSB, so a question is that what is the thickness of the crystals used in the measurements?

Minor issues:
1. The θ_k already appeared in the first paragraph, while the abbreviation MOKE (θ_k) appeared in the thrid paragraph;
2. "The sign of θ_c remains unchanged after thermal cycles in both sample 1 and 2, …", should be "… samples 1 and 2…";
3. Please carefully check the references because some references were already published rather than only on arXiv. Moreover, in ref. 34, delete the space among the letters of CsV_3Sb_5;

Reviewer #2:
Remarks to the Author:
C. Farhang et. al. employed both optical polarization rotation and Sagnac interferometer to investigate the time-reversal symmetry breaking (TRSB) in Kagome metal CsV_3Sb_5. They detected a large isotropic component θ_c (~1.4 mrad) and a smaller anisotropic one θ_p (~0.2 mrad) that emerged below T_{CDW} in optical rotation measurements. Interestingly, the change of

optical rotation $\Delta\theta$ was much smaller than θc under magnetic field, unlike the magneto-optic Kerr effect (MOKE) signal. They also confirmed the sample-dependent sign of θc by measuring another CsV3Sb5 sample. In θK measurement, they observed a small θK signal with a sharp drop below TCDW, which only appeared under magnetic field, indicating the Pauli paramagnetism nature. Based on these results, they distinguished the isotropic component θc and θK. The authors argued that the isotropic polarization component θc was not MOKE but originated from a new intertwined order. Their experimental method is careful and rigorous, and their conclusion seems convincing. I have some questions as follows:

1. In Fig. 1(d), the horizontal light blue and red dashed lines indicate the θc and θp values, respectively. It would be clearer if these lines were only shown below 94 K
2. As shown in Fig. 2(a), there is a polarizer in the Sagnac interferometer. In general, when a laser beam passes through a polarizer, it loses its initial polarization information and becomes linearly polarized along the transmission axis. How can the phase difference Δφ be measured when the reflected beam crosses the polarizer?
3. The scanning images of reflected optical power P0 show obvious position dependence (from light blue color (~10 μW) to red color (~25 μW)). Is this caused by the uneven sample surface? How can the accuracy of θK measurement be ensured in this case?
4. There are three kinds of signals detected (θc, θp, and θK), where θp and θK can be attributed to 2-fold-rotation-symmetry structural transition and TRSB, respectively. The origin of θc signal, however, remains unclear. Can the authors provide more in-depth discussion or analysis on this? This would make the paper more refined.

In general, the authors have performed comprehensive optical experiments to distinguish the TRSB in CsV3Sb5 and to unravel the origin of the discrepancy between the optical rotation and other experimental methods. The presented results are novel and interesting. I recommend publishing this work in Nature Communications after addressing the above concerns.

Dear referees:

Thank you for your professional assessments and insightful suggestions. Attached below are the summary of changes and point-to-point responses to comments and questions.

-Jing Xia, on behalf of all authors

Summary of Changes

Changes in the main text are highlighted in yellow. There is no change in the supplementary information.

1. The abstract is shortened to within the 150 words limit.
2. Section titles such as "Results" and "Discussion" are added.
3. A "Methods" section is added by copying relevant parts from the supplementary information.
4. The dashed lines in Fig. 1(d) are shortened for clarity following the referee suggestion.
5. In the first paragraph on page 4, a sentence regarding reference 24 has been moved forward, and grammar mistakes are fixed following the referee comments.
6. In the "Reference", arxiv postings that have been recently published or accepted for publication have been updated, following referee comments.

Response to Referees

(Repones are in blue for clarity.)

Reviewer #1 (Remarks to the Author):

The authors reported herein their optical rotation and polar MOKE measurements at a specific wavelength of 1550 nm on CsV_3Sb_5 crystals. They found a giant polarization independent isotropic components θ_c below the CDW temperature which do not respond to external magnetic field but flip the sign between samples. Meanwhile, the MOKE signal θ_k was found to be negligible. The authors argued that the observations indicate a weak link between the isotropic rotation and the experimentally observed time-reversal symmetry breaking by STM and other experiments. The authors attributed the isotropic rotation θ_c to either intertwined order or a novel hybrid phenomenon from the complex intertwining of the various orders in the CDW state, rather than time-reversal symmetry breaking, anisotropy or chiral order. This work not only clarified the discrepancy among various previous experiments reported different θ_c and θ_k and the relation between isotropic rotation and time-reversal symmetry breaking, but also clearly demonstrated the long-sought isotropic specular optical rotation in a solid.
This is an in-time and rigorous work that could stimulate much interest in further understanding the time-reversal symmetry breaking in these kagome compounds and the related intriguing physics. It may warrant its publication in Nature communications after addressing the following concerns:

We thank the reviewer for his/her professional assessments and suggestions.

1． The isotropic components θ_c show sample dependence, which in sample 2 are even opposite to that found in sample 1. The authors said "This can be explained if multi-domains of "sample 1" type and "sample 2" type are present in their sample, which will lead to location-dependent values of $\pm\theta_c$, and even intermediate values for sub-wavelength (1 μm) domains. It may also explain why their measured θ_c is one order of magnitude smaller than possible "single domain" samples in this work and in another 800 nm experiment where θ_c of the same sign and similar sizes were found in two CsV_3Sb_5 samples". It is somewhat astonishing that the different crystals can have so apparent difference in θ_c. If the difference is actually related to the domain sizes, it will be very crucial to characterize the domains of the crystals. Had

the authors checked the domain sizes in their two crystals? A direct comparison of the domains of the two samples would be more straightforward for the readers to understand this argument.

We have measured polarization rotation at various spots but found no evidence of θ_C domains in the two samples that we have received and studied. The figure below shows raw θ_T polar plots at various locations in both samples at the base temperature of ~ 4 *K*. Panel (a) is for sample 1 and panel (b) is for sample 2. The four spots for each sample are roughly in the middle of the four quadrants of the sample surface. Although we can't do scanning imaging for θ_T due to the lack of a powerful enough scanner for the bulky free-space optics, these manually positioned measurements are almost location-independent, leading us to conclude that polarization-rotation wise, the two samples we have are of a single θ_C domain.

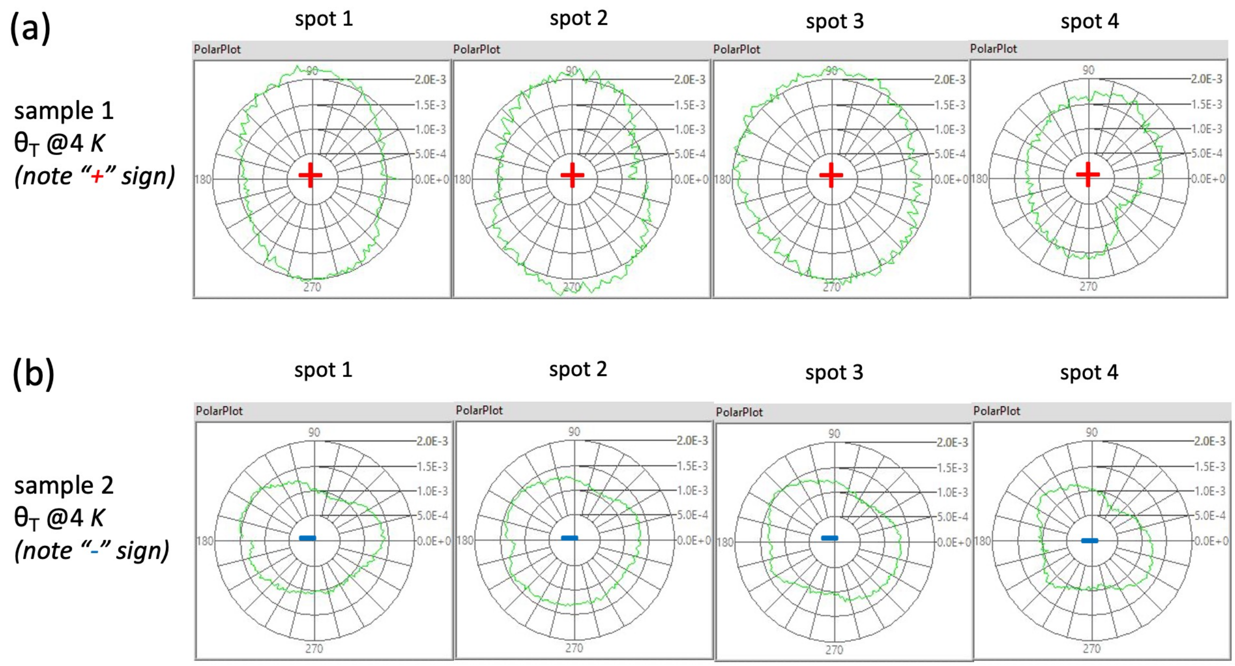

Comparing this with the two 800 nm polarization rotation measurements: reference 24 reported θ_C of ~0.5 mrad, which roughly agrees with our value of ~1 mrad, considering the wavelength difference; however, reference 23 found a θ_C of ~0.05 mrad, which is roughly 10 times smaller than both our result and that of reference 24. Like the referee, I was puzzled by why reference 23's value is so different from others. Then I noticed that reference 23 is the only work that has observed opposite signs of θ_C at different locations in the same sample. This indicates θ_C domain formation in reference 23 and could explain the reported smaller θ_C.

The next question is: why would the sample in reference 23 break into θ_C domains? I have a few speculations: growth condition, sample handling, and strain in sample mounting. In a free-space polarization rotation setup, usually scanning imaging is accomplished by mechanically moving the much lighter sample with a cryogenic scanning stage. Therefore, it is possible that in reference 23, which is the only work with scanning imaging of polarization rotation, the associated complexity with mounting the sample onto a cryogenic scanner stage has introduced strain, which might lead to θ_C domains. Of course, these are all speculative. And a definite answer may be obtained after communicating with the authors of reference 23 to examine and compare their experimental details.

2. On how many places of one crystal the authors measured the data? When measured different places of the same piece of crystal, are the results reported herein reproducible?

The results at various locations in the same crystal are reproducible for both MOKE and polarization rotations.

For Sagnac MOKE (θ_K), we were able to perform imaging by scanning the fiber optics. These scans contain tens of thousands of locations and are shown in the main text Fig.2 c, d for sample 1 and Fig.3 d, e for sample 2. The MOKE results are found to be location independent.

For polarization rotation (θ_T), although we couldn't scan the bulk optics for imaging due to the lack of a powerful enough scanner, I was able to manually move the bulk optical setup and measure at a few locations in each sample. The results are shown in the figure above (in the answer to question 1). The isotropic component θ_C is of the same sign and of similar sizes in the same sample. The anisotropic component θ_P may have different orientations of the principal axis at different locations, like what was reported as the "nematic domains" in reference 23.

3. It is well known that loss of surface Cs tends to occur in CsV3Sb5, which can induce hole doping effect but could be very different in different samples. Will this bring influences on optical rotation and polar MOKE measurements?

The crystals were cleaved perpendicular to the c-axis to expose optically flat areas and were mounted to the sample stage right before pumping to vacuum. The Sagnac and polarization rotation measurements were then performed without the sample leaving the cryostat. Therefore, there was perhaps a maximum of 10 minutes when the fresh surface was exposed to air and could lose surface Cs. According to the authors who grew the crystals, this amount of time is too short to have a significant loss of Cs.

For a minuscule amount of loss of surface Cs, the air-exposure time between cleavage and vacuum in our experiments is comparable to or shorter than prior experiments on CVS (optical, muSR, transport, etc.), except for STM and ARPES where cleavage was performed inside the vacuum chamber. This means the samples measured here are quite comparable to those in previous optical, muSR and transport experiments in terms of surface Cs. The point of this paper is to measure and compare both MOKE and polarization rotation in the same sample without breaking the vacuum to eliminate the effects of sample dependence and degradation.

Could a very Cs-deficient crystal, such as one can perhaps intentionally grow, have a different optical rotation and MOKE response? I would guess that due to the change of DOS (hole doping), the Pauli paramagnetic response would change, hence the MOKE signal under a magnetic field. For the optical rotation, since we don't fully understand its mechanism, I don't want to speculate, but it will be an interesting future work to carry out.

4. After cleaved the crystals for measurements, how the authors guaranteed the crystal quality during measurements? Will the crystals be degenerated during measurements?

The crystals were cleaved perpendicular to the c-axis to expose optically flat areas and were mounted to the sample stage right before pumping to vacuum. The air-exposure time is less than 10 minutes. The Sagnac and polarization rotation measurements were then performed within a few days without the sample leaving the UHV environment of the cryostat. These measures guarantee that there was little degradation during and between MOKE and polarization rotation measurements.

5. The time-reversal symmetry breaking is tightly related to the 3D CDW in CsV3Sb5 which is

thickness dependent, where the change of CDW phase may have influence on the TRSB, so a question is that what is the thickness of the crystals used in the measurements?

For sample 1, the thickness is 0.2 mm. For sample 2, the thickness is 0.3 mm.

Minor issues:
1. The θk already appeared in the first paragraph, while the abbreviation MOKE (θk) appeared in the thrid paragraph;

We thank the referee for pointing this out. In paragraph 3, we have deleted the redundant abbreviations "(θ_T)" and "(θ_K)".

2. "The sign of θc remains unchanged after thermal cycles in both sample 1 and 2, …", should be "… samples 1 and 2…";

We thank the referee for pointing this out. It is fixed.

3. Please carefully check the references because some references were already published rather than only on arXiv. Moreover, in ref. 34, delete the space among the letters of CsV3Sb5;

We thank the referee for pointing this out. It is fixed.

Reviewer #2 (Remarks to the Author):
C. Farhang et. al. employed both optical polarization rotation and Sagnac interferometer to investigate the time-reversal symmetry breaking (TRSB) in Kagome metal CsV3Sb5. They detected a large isotropic component θc (~1.4 mrad) and a smaller anisotropic one θp (~0.2 mrad) that emerged below TCDW in optical rotation measurements. Interestingly, the change of optical rotation $\Delta\theta$ was much smaller than θc under magnetic field, unlike the magneto-optic Kerr effect (MOKE) signal. They also confirmed the sample-dependent sign of θc by measuring another CsV3Sb5 sample. In θK measurement, they observed a small θK signal with a sharp drop below TCDW, which only appeared under magnetic field, indicating the Pauli paramagnetism nature. Based on these results, they distinguished the isotropic component θc and θK. The authors argued that the isotropic polarization component θc was not MOKE but originated from a new intertwined order. Their experimental method is careful and rigorous, and their conclusion seems convincing. I have some questions as follows:

We thank the reviewer for his/her professional assessments and suggestions.

1. In Fig. 1(d), the horizontal light blue and red dashed lines indicate the θc and θp values, respectively. It would be clearer if these lines were only shown below 94 K

We thank the referee for this suggestion. Fig. 1(d) has been updated.

2. As shown in Fig. 2(a), there is a polarizer in the Sagnac interferometer. In general, when a laser beam passes through a polarizer, it loses its initial polarization information and becomes linearly polarized along the transmission axis. How can the phase difference Δφ be measured when the reflected beam crosses the polarizer?

The details of the Sagnac interferometer are explained on page 2 and 3 of the supplementary information. Regarding the polarizer, its function is to split and then combine the two light beams for interference: The fiber optics used in the interferometer are "polarization maintaining", allowing only two orthogonal linear polarization eigen components along the so-called "fast" and "slow" axes of the fiber (In the free-space region near the sample, a quarter-wave plate converts these two linear polarizations into opposite circular polarizations; and after sample reflection the same quarter wave plate converts them back into switched linear polarizations). The instrument is designed such that the "interference" occurs between these two linear polarization components. The polarizer, whose polarization axis is aligned at $45^o(-45^o)$ to the fast(slow) axis of the fiber, serves two purposes. When the lights travel through it to the sample, the polarizer splits roughly equal amounts of polarizations onto the "fast" and "slow" axes of the fiber. When the light returns from the sample, the two linearly polarized components in the fiber, now switched, have a phase difference $\Delta\varphi = 2\theta_K$, which we want to measure by interference. This is accomplished by passing the returned light through the 45° polarizer a second time: the polarizer forces both linearly polarized components in the fiber to "project" along the polarizer's transmission axis and hence "interfere". $\Delta\varphi$ is then extracted from the detected optical power (interference intensity).

3. The scanning images of reflected optical power P0 show obvious position dependence (from light blue color (~10 μW) to red color (~25 μW)). Is this caused by the uneven sample surface? How can the accuracy of θK measurement be ensured in this case?

It is not uncommon to see 50% change of reflected power in a fiber Sagnac interferometer on a nominally flat surface. The fiber in the Sagnac interferometer is a "single mode" fiber that only couples to light of almost strictly normal incidence, i.e. reflected from an optically flat surface. A tilt of half a degree would reduce the returned optical power to essentially zero. From the changes in P_0 we estimate the sample surface is a bit curved by about a quarter of a degree, or 2 microns across the sample. The measured θ_K is not affected by such a small curvature. In fact, this "single mode" fiber works as an angular spatial filter to ensure normal incidence ("polar" Kerr geometry). The measured θ_K is not affected by the 50% change of the reflected optical power (P_0) either. This is because θ_K is calculated by the ratio between the first harmonics power P_1 and second harmonics power P_2, both scales with P_0 and thus cancel out. Details of θ_K calculation can be found on page 2 and 3 of the supplementary information.

4. There are three kinds of signals detected (θc, θp, and θK), where θp and θK can be attributed to 2-fold-rotation-symmetry structural transition and TRSB, respectively. The origin of θc signal, however, remains unclear. Can the authors provide more in-depth discussion or analysis on this? This would make the paper more refined.

Although the existence of θ_K in CsV3Sb5 has been reported experimentally by references 23, 24 and this work, its theoretical origin is not yet clear. And it may ultimately be related to the proper material equation for the electrodynamics of nonlocal optical response in CsV3Sb5. In principle, without MOKE, a polarization rotation proportional to $\langle\Psi_g|m_x|\Psi_e\rangle\langle\Psi_g|x|\Psi_e\rangle + \langle\Psi_g|m_y|\Psi_e\rangle\langle\Psi_g|y|\Psi_e\rangle$ can still exist, where $\Psi_g(\Psi_e)$ are ground(excited states), m_x/m_y are magnetic moments along x and y directions. Historically there were "Casimir" and "Landau-Lifshitz" approaches to calculating the polarization rotation in an optically non-local media. In the paper by Bungay et. al. (Phys. Rev. B 47, 11730–11735 (1993)), it was shown that the two approaches are equivalent by considering all the derivatives of the nonlocal susceptibilities, and that the specular rotation can be viewed as "the complex conjugated phenomenon" with respect to optical activity in transmission. But how is this relevant to CsV3Sb5? What are the non-local responses in the CDW

state (and potentially the loop currents)? We don't know the answers yet. And I think it is too speculative at this stage to discuss them in the paper.

In general, the authors have performed comprehensive optical experiments to distinguish the TRSB in CsV3Sb5 and to unravel the origin of the discrepancy between the optical rotation and other experimental methods. The presented results are novel and interesting. I recommend publishing this work in Nature Communications after addressing the above concerns.

We thank the reviewer again for his/her professional assessments and suggestions.

Reviewers' Comments:

Reviewer #1:
Remarks to the Author:
I am satisfied with the responses and revisions made by the authors. Therefore, I would like to recommend a publication of this work in Nature Communications.

Reviewer #2:
Remarks to the Author:
I am satisfied with the response and revision. I recommend acceptance of the manuscript for publication in Nature Communications.

REVIEWERS' COMMENTS

Reviewer #1 (Remarks to the Author):

I am satisfied with the responses and revisions made by the authors. Therefore, I would like to recommend a publication of this work in Nature Communications.

Reviewer #2 (Remarks to the Author):

I am satisfied with the response and revision. I recommend acceptance of the manuscript for publication in Nature Communications.

We thank referee 1 and 2 for their professional assessments and insightful suggestions.

-Jing Xia on behalf of all authors
